# Treatment of systemic lupus erythemus overlap syndrome with autoimmune hepatitis using a combination of glucocorticoids and immunosuppressive agents: Case report

**DOI:** 10.1097/MD.0000000000043570

**Published:** 2025-08-01

**Authors:** Zhijie Li, Zhijian Zha, Suhua Zhang, Tingyu Jiang, Yang Wu, Wei Lu, Yunhai Li, Jianzhong Liu, Lianmei Lin

**Affiliations:** a Hubei University of Chinese Medicine, Wuhan, China; b Jingzhou Traditional Chinese Medicine Hospital, Jingzhou, China; c Hubei Shizhen Laboratory, Wuhan, China; d Hubei Provincial Hospital of Traditional Chinese Medicine, Affiliated Hospital of Hubei University of Chinese Medicine, Wuhan, China; e Hubei Key Laboratory of Liver and Kidney Research and Application of Traditional Chinese Medicine, Wuhan, China.

**Keywords:** autoimmune hepatitis, glucocorticoids, hepatic injury, immunosuppressive agents, systemic lupus erythematosus

## Abstract

**Rationale:**

Systemic lupus erythematosus (SLE) and autoimmune hepatitis (AIH) are distinct autoimmune disorders whose concurrent presentation (overlap syndrome) poses significant diagnostic and therapeutic challenges. While SLE-associated liver involvement is recognized, true SLE-AIH overlap is rare and lacks established management guidelines. This report details the successful management of such a case to contribute insights into this complex clinical scenario.

**Patient concerns:**

A 35-year-old male presented with a 1-year history of intermittent facial and body rashes accompanied by significant weight loss (approximately 10 kg). Over the preceding half-month, he developed signs of liver injury, including fatigue, cold intolerance, loss of appetite, and generalized weakness.

**Diagnoses:**

Comprehensive diagnostic evaluation, cutaneous manifestations: facial and generalized skin rash, hematologic abnormalities: cytopenia, including serological testing (positive antinuclear antibody [1:1000], anti-SS-A antibodies, elevated immunoglobulin G, rheumatoid factor, erythrocyte sedimentation rate, C-reactive protein, decreased complement C3 and C4 levels), liver function tests (persistently elevated alanine aminotransferase, aspartate aminotransferase, gamma-glutamyl transferase, alkaline phosphatase, globulin), and imaging (uneven liver texture/density on ultrasound/computed tomography), confirmed a diagnosis of SLE complicated by AIH. The diagnosis of SLE meets both the 1997 American College of Rheumatology classification criteria and the 2012 Systemic Lupus International Collaborating Clinics revised criteria. AIH diagnosis was supported by both the 1990 (score=16) and 2008 (score=7) International Autoimmune Hepatitis Group (IAIHG) scoring systems. Infectious and other common causes of liver injury were excluded.

**Interventions:**

Initial management focused on controlling liver injury using intravenous and oral hepatoprotective agents (inosine, bicyclol, silibinin, compound glycyrrhizin, magnesium isoglycyrrhizinate). High-dose intravenous glucocorticoids (dexamethasone 5–10 mg daily) were initiated concurrently to suppress autoimmune activity. Upon stabilization of liver function, treatment transitioned to oral prednisone (30 mg daily) combined with the immunosuppressant leflunomide (10 mg daily). Glucocorticoids were subsequently tapered strictly according to plan, while leflunomide monotherapy was maintained long-term. Adjunctive therapies included calcium and gastric protection.

**Outcomes:**

Liver function tests normalized following the combined hepatoprotective and glucocorticoid therapy. The introduction and maintenance of leflunomide, alongside glucocorticoid tapering, effectively controlled SLE disease activity. At a 2-year follow-up, the patient remained clinically stable with normalized liver function, no recurrence of severe symptoms, and a good quality of life.

**Lessons:**

This case highlights the efficacy of a sequential treatment approach combining glucocorticoids and immunosuppressants (specifically leflunomide) for managing SLE-AIH overlap syndrome. Early diagnosis using established criteria is crucial. Multidisciplinary collaboration is essential. Long-term leflunomide monotherapy demonstrated sustained remission in this patient, although vigilance for adverse effects (e.g., hepatotoxicity) and disease flare is necessary. The absence of specific guidelines for this overlap syndrome underscores the need for individualized treatment strategies based on SLE and AIH management principles.

## 1. Introduction

Systemic lupus erythematosus (SLE) is a complex, chronic autoimmune disorder manifesting as a diffuse connective tissue disease with a predilection for multi-systemic involvement. It is typified by immunologically mediated inflammation, affecting a wide array of organs and tissues, and is characterized by a constellation of clinical and immunological abnormalities. Owing to heterogeneity in various factors, reports on the global incidence and prevalence of SLE exhibit considerable variability. Epidemiological studies have documented an incidence of SLE among Asian populations that spans a range of 2.8 to 8.6 cases per 100,000 person-years. In terms of survival, the reported rates at 1, 5, 10, and 15 years post-diagnosis exhibit a spectrum from 93.7% to 98.4%, 80.4% to 98.6%, 56.5% to 98.2%, and 31.7% to 88.8%, respectively, highlighting the variability in outcomes within this cohort.^[1]^ Autoimmune hepatitis (AIH) represents a condition wherein an aberrant autoimmune response culminates in inflammation of the hepatic parenchyma, specifically targeting hepatocytes. This pathology is distinguished by a constellation of biochemical and serological hallmarks including, but not limited to, heightened serum aminotransferase concentrations, the presence of autoantibodies in the serum, hypergammaglobulinemia predominantly involving increased immunoglobulin G (IgG) titers, alongside the hallmark histopathological feature known as interface hepatitis.^[2]^ According to epidemiological research, AIH exhibits a propensity toward onset in individuals within the age bracket of 50 to 60 years, with a striking gender disparity favoring females over males, as evidenced by male-to-female incidence ratios ranging from 1:4 to 1:6. In the context of European nations, AIH’s point prevalence has been quantified to fall within the interval of 10 to 25 occurrences per 100,000 populace. Notably, statistical data from New Zealand for the temporal segment encompassing the years 2014 through 2016 unveiled a prevalence figure amounting to 2.39 cases per 100,000 residents.^[3]^ The point prevalence rate in Japan for 2016 was 23.9 per 100,000 people.^[4]^ A single-center, retrospective observation study executed within China encompassed a cohort of 1020 patients suffering from AIH. The investigation disclosed that the modal age at diagnosis clustered around 55 years, albeit with considerable variation extending from early childhood to advanced age (spanning 6–82 years). Additionally, the demographic profile highlighted a pronounced female preponderance, encapsulated by a sex ratio delineating 1 male for every 5 affected females.^[5]^ Over the past 2 decades, there has been a globally recognized trend of increasing incidence of AIH.^[6]^ Clinicians have extensively documented overlap syndromes, characterized by the concomitant manifestation of SLE juxtaposed against a backdrop of systemic sclerosis, dermatomyositis/polymyositis, Sjögren syndrome, and rheumatoid arthritis. Despite this abundance, instances detailing the comorbidity of SLE coupled with AIH remain relatively scarce within the annals of medical reporting.

Liver injury in patients can result from various causes including lupus hepatitis induced by SLE itself, AIH, as well as drug-induced hepatitis caused by immunosuppressants used to control the disease condition. Diagnosing and managing the condition becomes quite challenging when SLE overlaps with AIH. Here, we present a patient with SLE overlaps with AIH showing discrete dermatological alterations on the face and liver dysfunction, and achieved the stable condition during the 2-year-follow-period with a combination therapy comprising glucocorticoids and immunosuppressants.

## 2. Diagnosis and treatment process

### 2.1. Case presentation

#### 2.1.1. Patient information

Herein, we report the case of a 35-year-old male patient who presented at Jingzhou Traditional Chinese Medicine Hospital and was diagnosed with SLE in conjunction with AIH. The patient’s main symptoms included intermittent rashes on the face and body along with weight loss for 1 year, accompanied by signs of liver injury for the past half month. The patient had rust-colored urine without frequency, urgency, and dysuria. His medical and family histories are unremarkable, and he has no known drug or food allergies.

#### 2.1.2. Current illness history

In March 2022, seeking relevant treatment, the patient visited our outpatient department at Jingzhou Traditional Chinese Medicine Hospital. A complete blood count revealed: leukocytes 2.65 × 10^9^/L↓, lymphocyte percentage 16.9%↑, and neutrophil percentage 45.5%↓; liver function tests showed alanine aminotransferase (ALT) 103.9 IU/L↑ and aspartate aminotransferase (AST) 68.2 IU/L↑. Following this, the patient received oral hepato-protective medication (details unspecified). Upon reexamination on March 8, liver functions returned to normal levels, leading to discontinuation of medication.

On July 28, 2022, the patient presented with fever, fatigue, cold intolerance, reaching up to 39 °C, loss of appetite, and thus sought emergency care at Jingzhou No. 1 People’s Hospital. Complete blood cell analysis indicated a lymphocyte percentage of 12.70%↓, neutrophil percentage of 73.90%↑, highly sensitive C-reactive protein (CRP) 133.80 mg/L↑; liver and kidney function tests revealed: direct bilirubin 9.4 μmol/L↑, ALT 52.3 IU/L↑, AST 56.1 IU/L ↑, alkaline phosphatase 166.8 U/L↑, γ-glutamyl transferase 129.8 U/L↑; urinalysis showed brownish coloration, bile pigments at 1+, protein in urine at 2+, urobilinogen at 1+. After receiving anti-infective and hepato-protective treatments, the aforementioned symptoms persisted along with generalized weakness. In recent months, there has been a weight loss of approximately 10 kg, prompting admission to our Gastroenterology Department.

On August 4, 2022, examination at our hospital: color Doppler ultrasound of the liver, gallbladder, spleen, pancreas, and portal vein: uneven texture of the liver. Contrast-enhanced abdominal CT with 3D reconstruction: uneven decrease in liver tissue density, urinalysis: hemolyzed RBCs 1.2 cells/μL↑, albumin 80 mg/L↑, protein/creatinine ratio 0.30 g/gCr↑, albumin/creatinine ratio 80 mg/gCr↑; liver function: ALT 78.1 IU/L↑, AST 84.0 IU/L↑, γ-glutamyl transferase 164.1 IU/L↑. CRP 75.76 mg/L↑; erythrocyte sedimentation: erythrocyte sedimentation rate (ESR) 71 mm/h↑; rheumatoid factor 28.4 IU/mL↑; immunological panel: immunoglobulin A 4.01 g/L↑, IgG 16.33 g/L↑, complement C3 0.761 g/L↓, complement C4 0.150 g/L↓; antinuclear antibody test: positive↑; antinuclear antibody titer: 1:1000↑; antinuclear antibody spectrum 17 items: anti-SS-A60 antibody positive (+), anti-SS-A52 antibody positive (+), others negative; serum ferritin: ferritin 1169.10 µg/L↑; AIH 8 items: anti-RO-52 antibody positive (+), others negative. No abnormalities were detected in the 3-part hepatitis B system, hepatitis C antibodies, syphilis antibodies, or human immunodeficiency virus antibodies. Additional laboratory examinations revealed no remarkable deviations from normal. During this period, anti-inflammatory and hepato-protective treatments were administered as symptomatic management.

On August 14th, reexamination of liver function: 5-nucleotidase 26.9 U/L↑, cholinesterase 3444 U/L↓, globulin 43.10 g/L↑, ALT 144.5 IU/L↑, AST 104.6 IU/L↑, alkaline phosphatase 202 IU/L↑, γ-glutamyl transferase 137.3 IU/L↑, and albumin/globulin ratio 0.82↓. As the patient’s condition remained uncontrolled, admission to our hospital’s Rheumatology and Immunology department occurred on August 22, 2022.

#### 2.1.3. Clinical findings

Upon examination, the patient displayed erythematous lesions interspersed with vesicular eruptions prominently located on the facial area, bilateral hands, and superior extremities. Subsequent to the resolution phase of these cutaneous findings where rashes subsided and blisters degenerated into crusts, post-inflammatory pigmentation alongside desquamation became evident within these regions.

After admission, relevant auxiliary examinations were completed: on August 23, 2022, full blood cell analysis showed leukocytes at 3.53 × 10^9^/L↓, erythrocytes at 3.74 × 10^12^/L↓, monocyte percentage at 12.1%↑, absolute lymphocyte count at 1.05 × 10^9^/L↓, mean red cell volume at 100.9 fL↑, and red blood cell distribution at 50.9 fL↑; rheumatoid factor at 32.5 IU/mL↑; ESR 90 mm/h↑; liver function tests indicated: ALT 82.5 U/L↑, AST 66.4 IU/L↑, alkaline phosphatase 197 IU/L↑, γ-glutamyl transferase 95.1 U/L↑, 5’-nucleotidase 19.6 U/L↑, cholinesterase 3135 U/L↓, globulin 44.7 g/L↑; albumin/globulin ratio at 0.83↓; immunological panel showed immunoglobulin A 4.00 g/L (↑), IgG 21.36 g/L (↑); antineutrophil cytoplasmic antibody perinuclear type positive (+), and antineutrophil cytoplasmic antibody cytoplasmic type: negative.

### 2.2. Timeline

After admission, the diagnosis was made as systemic lupus erythematosus complicated with AIH. With the exclusion of diseases like tumors and infectious diseases, on August 23, 2022, the patient received glucocorticoid treatment to control the condition (intravenous drip of dexamethasone sodium phosphate injection at a dose of 5 mg once daily), treatment for liver function restoration (intravenous drip of inosine injection at a dose of 0.2 g once daily plus oral administration of bicyclol tablets at a dose of 25 mg 3 times daily), along with adjunctive treatments such as calcium supplementation and gastric mucosa protection.

On August 26, 2022, re-examine relevant biochemical indicators, ESR: 68 mm/h↑; liver function: ALT 128.7 IU/L↑, AST 101.0 IU/L↑, γ-glutamyl transferase 104.4 IU/L↑; globulin42.3 g/L↑. Therefore, the dose of glucocorticoids was increased (dexamethasone injection 10 mg, intravenous drip, once a day) to control the disease, and the liver-protecting treatment was strengthened (silibinin capsules 105 mg, oral administration, 3 times a day; compound glycyrrhizin capsules 75 mg, oral administration, 3 times a day).

On August 30, 2022, re-examine relevant biochemical indicators, ESR: 45 mm/h↑; liver function: ALT 134.7 IU/L↑, AST 118.7 IU/L↑, γ-glutamyl transferase 139.4 IU/L↑, globulin 41.3 g/L↑; no obvious abnormalities were found in electrolytes. The patient did not report any special discomfort. The liver-protecting treatment was adjusted to a quadruple therapy (magnesium isoglycyrrhizinate injection 1.0 g,s intravenous drip, once a day, and the other 3 drugs, namely glucurolactone tablets, compound glycyrrhizin capsules, and silibinin capsules, were taken in the same way as before).

On September 4, 2022, re-examine relevant biochemical indicators, ESR was 52 mm/h↑; liver function: ALT 79.4 IU/L↑, AST 53.5 IU/↑, γ-glutamyl transferase 155.1 IU/L↑, globulin 38.80 g/L↑. According to relevant auxiliary examinations, the patient’s condition gradually tended to be stable, and the treatment plan was adjusted: glucocorticoids (the daily intravenous drip dose of dexamethasone sodium phosphate injection was reduced from 10–5 mg), and the rest of the treatment plan remained unchanged.

**Figure 1.**
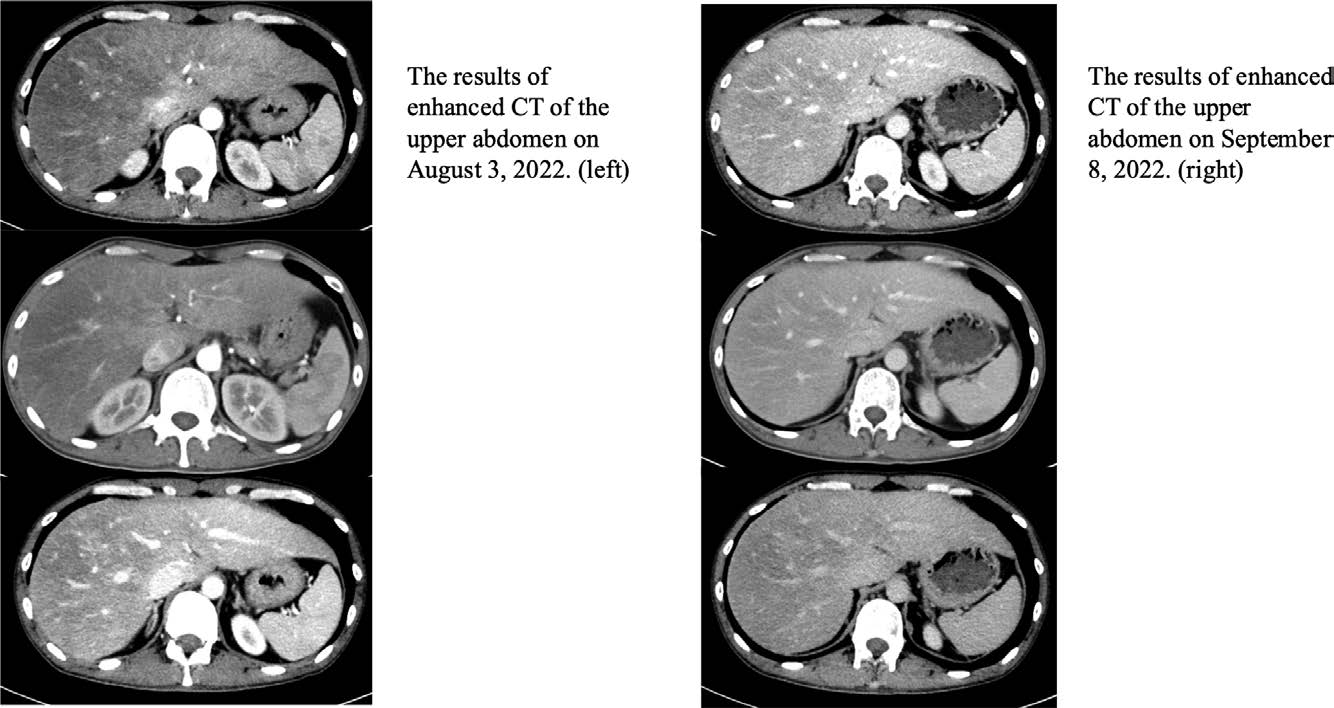
Enhanced CT scans of patients before and after treatment.

On September 8, 2022, re-examine relevant biochemical indicators, complete blood count analysis demonstrates normalization of both erythrocyte and leukocyte parameters. ESR 70 mm/h↑; liver function: ALT 69.3 IU/L↑, AST 48.9 IU/L↑, γ-glutamyl transferase 160.6 IU/L↑, globulin 38.1g/L↑. Plain and enhanced CT scan of the upper abdomen: the density of the liver parenchyma is unevenly decreased. Compared with the imaging results on August 4, the interface hepatitis has significantly improved (Fig. 1).

On September 9th, in order to further control the disease and reduce the toxic and side effects brought by the use of glucocorticoids, the glucocorticoid regimen was adjusted as follows: stop using dexamethasone sodium phosphate injection and change to prednisone acetate tablets 30 mg, oral administration, once a day, and combine with immunosuppressants to control the disease (leflunomide tablets 10 mg, oral administration, once a day).

**Figure 2.**
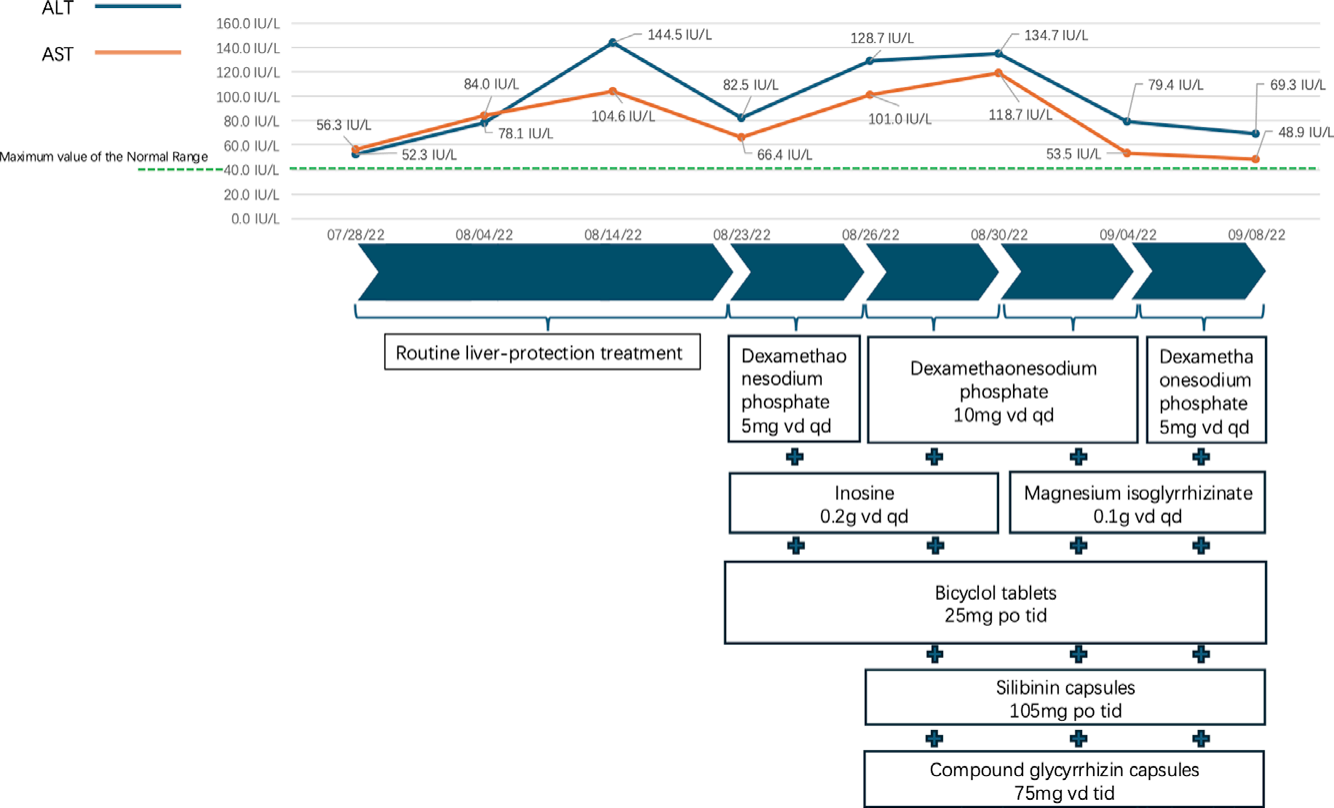
Changes in liver transaminases during the patient’s diagnosis and treatment process.

On September 10, 2022, the patient was arranged to go through the discharge procedures. Outside the hospital, the hormone dosage was strictly decreased step by step, and the immunosuppressant (leflunomide tablets 10 mg, taken orally once a day) was used at the regular dosage. On March 31, 2023, the patient had a re-examination of liver function in our outpatient department and no abnormality was found. During the subsequent follow-up, the patient did not complain of any discomfort and had a fairly good quality of life. Timeline of the relevant information is shown in Figure 2. The experimental data during the treatment process are presented in Table 1.

**Table 1:** Clinical laboratory of data form.

Time	Clinical laboratory of data form									
	Test Items	Parameter	Value		Unit	Interpretation				Reference
March 01, 2022	Complete blood count	Leukocytes	2.65		×10^9^/L	Low				4.0–11.0
		Lymphocyte percentage	16.9		%	Low				20–40
		Neutrophil percentage	45.5		%	Low				50–70
	Liver function tests	ALT	103.9		IU/L	High				10–40
		AST	68.2		IU/L	High				8–40
June 28, 2022	Complete blood count	Lymphocyte percentage	12.70		%	Low				20–40
		Neutrophil percentage	73.90		%	High				50–70
	Liver function tests	ALT	52.3		IU/L	High				10–40
		AST	56.1		IU/L	High				8–40
		Alkaline phosphatase	166.8		U/L	High				40–130
		γ-Glutamyl transferase	129.8		U/L	High				10–60
	CRP		133.80		mg/L	High				≤5
	Urinalysis	Brownish coloration								
		Bile pigments	1+							
		Protein in urine	2+							
		Urobilinogen	1+							
August 04, 2022	Liver function tests	ALT	78.1		IU/L	High				10–40
		AST	84.0		IU/L	High				10–40
		γ-Glutamyl transferase	164.1		U/L	High				10–60
	CRP		75.76		mg/L	High				≤5
	Urinalysis	Hemolyzed RBCs	1.2		cells/μL	High				
		Albumin	80		mg/L	High				<30
		Protein/creatinine ratio	0.30		g/gCr	High				<0.15
		Albumin/creatinine ratio	80		mg/gCr	High				<30
	ESR		71		mm/h	High				0–15
	RF		28.4		IU/mL	High				≤20
	Immunological panel	IgA	4.01		g/L	High				7.0–16.0
		IgG	16.33		g/L	High				0.7–4.0 g/L
		Complement C3	0.761		g/L	Low				0.9–1.8 g/L
		Complement C4	0.150		g/L	Low				0.1–0.4 g/L
	Antinuclear antibody test		Positive (+)			High				
	Antinuclear antibody titer		1:1000			High				
	Antinuclear antibody spectrum 17 items	Anti-SS-A60 antibody positive (+); anti-SS-A52 antibody positive (+); others negative								
	Autoimmune hepatitis 8 items	Anti-RO-52 antibody positive (+); others negative								
August 14, 2022	Liver function tests	ALT		144.5	IU/L			High	10–40	
		AST		104.6	IU/L		High			8–40
		Alkaline phosphatase		202	U/L		High			40–130
		γ-Glutamyl transferase		137.3	U/L		High			10–60
		5-Nucleotidase		26.9	U/L		High			2–17
		Cholinesterase		3444	U/L		Low			5000–12,000
		Globulin		43.10	g/L		High			20–35
		Albumin/globulin ratio		0.82			Low			1.2–2.2
August 23, 2022	Complete blood count	Leukocytes		3.53	×10^9^/L		Low			4.0–11.0
		Erythrocytes		3.74	×10^12^/L		Low			4.3–5.8
		Monocyte percentage		12.1	%		High			3–10
		Absolute lymphocyte count		1.05	×10^9^/L		Low			1.0–4.8
		Mean red cell volume		100.9	fL		High			80–100
		Red blood cell distribution		50.9	fL		High			39–46
	ESR			90	mm/h		High			0–15
	RF			32.5	IU/mL		High			≤20
	Liver function tests	ALT		82.5	IU/L		High			10–40
		AST		66.4	IU/L		High			8–40
		Alkaline phosphatase		197	U/L		High			40–130
		γ-Glutamyl transferase		95.1	U/L		High			10–60
August 23, 2022	Liver function tests	5-Nucleotidase		19.6	U/L		High			2–17
		Cholinesterase		3135	U/L		Low			5000–12,000
		Globulin		44.7	g/L		High			20–35
		Albumin/globulin ratio		0.83			Low			1.2–2.2
	Immunological panel	IgA		4.00	g/L		High			7.0–16.0
		IgG		21.36	g/L		High			0.7–4.0 g/L
	p-ANCA			Positive (+)						
	c-ANCA			Negative						
August 26, 2022	ESR			68	mm/h		High			0–15
	Liver function tests	ALT		128.7	IU/L		High			10–40
		AST		101.0	IU/L		High			8–40
		γ-Glutamyl transferase		104.4	U/L		High			10–60
		Globulin		42.30	g/L		High			20–35
August 30, 2022	ESR			45	mm/h		High			0–15
	Liver function tests	ALT		134.7	IU/L		High			10–40
		AST		118.7	IU/L		High			8–40
		γ-Glutamyl transferase		139.4	U/L		High			10–60
		Globulin		41.3	g/L		High			20–35
September 04, 2022	ESR			52	mm/h		High			0–15
	Liver function tests	ALT		79.4	IU/L		High			10–40
		AST		53.5	IU/L		High			8–40
		γ-Glutamyl transferase		155.1	U/L		High			10–60
		Globulin		38.80	g/L		High			20–35
September 04, 2022	ESR			70	mm/h		High			0–15
	Liver function tests	ALT		69.3	IU/L		High			10–40
		AST		48.9	IU/L		High			8–40
		γ-Glutamyl transferase		160.6	U/L		High			10–60
		Globulin		38.1	g/L		High			20–35

ALT = alanine aminotransferase, AST = aspartate aminotransferase, c-ANCA = antineutrophil cytoplasmic antibody cytoplasmic type, CRP = C-reactive protein, ESR = erythrocyte sedimentation rate; RF = rheumatoid factor, GTT = γ-glutamyl transferase, IgA = immunoglobulin A, IgG = immunoglobulin G, p-ANCA = antineutrophil cytoplasmic antibody perinuclear type.

## 3. Discussion

### 3.1. Difficulty 1: diagnosis

Clinically, diseases with abnormal liver function caused by autoimmune diseases can be roughly divided into systemic autoimmune diseases that themselves involve the liver (such as connective tissue diseases like SLE, systemic scleroderma, and Sjögren syndrome) and organ-specific autoimmune diseases (AIH, primary biliary cholangitis, primary sclerosing cholangitis).^[7]^ Since the pathogenesis of SLE and AIH has not been fully understood yet. The concurrent or sequential abnormal liver functions this time are all attributed to the abnormal immune state. Some studies have pointed out that 19.4% to 60% of SLE patients will experience liver damage at a certain stage of the disease development, mainly lupus hepatitis and drug-induced liver injury, but among them, the more severe liver damage is mostly caused by AIH.^[8,9]^ According to the comprehensive diagnostic scoring system for AIH formulated by the International Autoimmune Hepatitis Group (IAIHG) in 1990,^[10]^ the patient’s pre-treatment score was 16 points, so AIH could be clearly diagnosed; using the simplified diagnostic scoring system for AIH proposed by IAIHG in 2008,^[11]^ the patient’s score was 7 points (a score of ≥ 7 can clearly diagnose AIH), so the diagnosis of AIH is clear. Due to the high similarity in clinical manifestations and laboratory tests, it is very important to effectively distinguish lupus hepatitis from AIH during the diagnosis process, and there are differences in the treatment strategies and prognoses of the 2 diseases.^[12]^ SLE more commonly leads to end stage renal disease, AIH often leads to end stage liver disease.^[13]^ In the diagnosis of diseases, liver biopsy is a crucial test for differentiating AIH from non-specific lupus liver involvement in SLE patients.^[9,14]^ The liver histologic abnormalities in SLE include portal, periportal, or lobular hepatitis with or without necrosis, cholestasis, steatosis, small artery vasculitis, gran ulomatous hepatitis, nodular regenerative hyperplasia, and peliosis hepatitis. The characteristic histologic features of AIH are portal mononuclear infiltrates that can invade the limiting plate, and infiltrate into the surrounding lobule causing periportal piecemeal necrosis.^[12]^ However, in view of the patient’s will, this patient did not have a liver biopsy. Combined with the patient’s other auxiliary examinations and clinical manifestations, the relevant diagnostic criteria have been met, so the results of liver biopsy are not strongly required. However, in the clinical process, when the diagnosis between SLE and AIH is ambiguous, liver biopsy is still an important means to make a definite diagnosis.

### 3.2. Difficulty 2: therapeutic methods

At the beginning of hospitalization, the patient’s condition was not effectively relieved and the treatment goal was not achieved. Based on the 1999 report by the International Autoimmune Hepatitis Group: the overlap syndrome between systemic lupus erythematosus and AIH demonstrates prompt corticosteroid responsiveness, with concomitant improvement in hepatic dysfunction and systemic manifestations of SLE.^[10]^ According to the 2015 Consensus on Diagnosis and Management of Autoimmune Hepatitis issued by the Chinese Society of Hepatology, Chinese Medical Association, monotherapy with corticosteroids is recommended for AIH complicated by cytopenia.^[15]^ Evidence from Japanese clinical studies indicates that early high-dose intravenous glycyrrhizin-based preparations can mitigate progression in acute flares of AIH. Thus, prior to initiating targeted immunotherapy, these agents represent a safe and effective initial therapeutic approach for acute hepatitis of undetermined etiology.^[16]^ During the diagnosis and treatment process, a large-dose of glucocorticoid was used to control the condition while continuously strengthening the liver-protecting treatment, so that after the liver function damage was alleviated, combined immunosuppressants were added to control the disease. Per the 2021 Chinese Society of Hepatology, Chinese Medical Association Consensus on Diagnosis and Management of Autoimmune Hepatitis, dual-agent regimens should be prioritized to minimize corticosteroid exposure, with subsequent transitioning to maintenance immunosuppressive monotherapy.^[17]^ Notwithstanding guideline recommendations for azathioprine as first-line therapy, our multidisciplinary team has initiated adjunctive leflunomide therapy following comprehensive clinical assessment of the patient’s specific disease profile. During the subsequent follow-up process, the glucocorticoid was strictly tapered off gradually, and the use of immunosuppressant (Leflunomide tablets, 10 mg, orally, once a day) was maintained to stably control the condition, achieving relatively ideal curative effects. Clinically, the combined use of glucocorticoids and immunosuppressants aims to consolidate remission and prevent disease activity. When disease control is achieved, minimizing glucocorticoid exposure is also consistent with SLE management recommendations.^[18]^ A notable clinical observation in this patient’s surveillance period was 1-year sustained remission maintained solely through leflunomide monotherapy: a therapeutic approach not encompassed by current clinical guidelines. When looking back at the intervention methods in this case, through timely adjustment of the dosage of glucocorticoids, flexible adjustment of the liver-protecting treatment plan, and the subsequent use of combined immunosuppressants, the clinical efficacy was relatively ideal. However, throughout the entire diagnosis and treatment process, the author has not yet found clear guidelines, and is still in the exploratory stage. And regarding the dosage and duration of use of glucocorticoids, the selection of immunosuppressants, and the determination of the liver-protecting treatment plan, there are still no clear guidelines available for reference.

### 3.3. Difficulty 3: adverse effects associated with long-term use of glucocorticoids and immunosuppressants

Long-term use of glucocorticoids can lead to significant adverse effects. Beyond the commonly observed Cushingoid features, glucocorticoids can also exacerbate osteoporosis, leading to related bone diseases. Furthermore, their use is associated with the development of type 2 diabetes, cataracts, hypertension, infections (including the reactivation of latent tuberculosis), and psychiatric disorders.^[17]^ Advise the patient to supplement with calcium and vitamin D while on glucocorticoid therapy. Immunosuppressants inherently carry potential toxicities when administered long-term. Leflunomide tablets, as employed in this case, notably pose risks of hepatotoxicity, bone marrow suppression, and gastrointestinal reactions. Hence, close and regular monitoring of blood counts and periodic bone density assessments are essential. A point requiring particular attention is distinguishing drug-induced liver injury from hepatic damage caused by a flare of the underlying disease when liver impairment occurs: a significant clinical challenge. Acute-phase reactants (ESR and CRP) can provide some indication of disease activity and extent of damage, serving as reference markers to help distinguish drug-induced liver injury from disease relapse,^[19,20]^ concurrently, vigilance for infections and other inflammatory conditions is essential. As clinicians, we must select optimal treatment strategies to enhance patient survival rates and quality of life. However, evidence-based guidelines and recommendations for managing such overlap syndromes are notably absent at present.

### 3.4. Insights and experiences

The author’s experience is that for the treatment of liver damage in such patients, the rheumatology and immunology department should cooperate with relevant departments such as hepatology and surgery to carry out multidisciplinary diagnosis and treatment as early as possible, and conduct multiple evaluations and repeated interventions during the patient’s diagnosis and treatment process to control the condition as early as possible, avoid damage to multiple organs such as blood vessels, heart, lungs, and kidneys and life-threatening situations, and reduce the risk of disease progression, in order to improve the quality of life of patients.

## 4. Conclusions

This case documents a complex clinical presentation of AIH overlapping with SLE. It emphasizes maintaining heightened clinical vigilance to achieve definitive early diagnosis and stringent disease control during patient management. Reciprocally, this demands clinicians possess exceptional professional competence and extensive clinical experience: capabilities that critically enable improved disease prognosis and optimal patient outcomes. A key limitation observed during the development of this case report is the scarcity of scientific literature and absence of clinical guidelines specific to AIH–SLE overlap syndrome. Consequently, current management primarily integrates recommendations for both AIH and SLE. However, pharmacotherapeutic regimens delivering optimal efficacy for treating AIH–SLE overlap remain unestablished.

## Acknowledgments

We thank the patient and his family for participating in this study.

## Author contributions

**Conceptualization:** Lianmei Lin, Jianzhong Liu.

**Data curation:** Suhua Zhang, Tingyu Jiang.

**Formal analysis:** Wei Lu.

**Investigation:** Yang Wu.

**Supervision:** Yunhai Li.

**Visualization:** Zhijie Li.

**Writing – original draft:** Zhijie Li.

**Writing – review & editing:** Zhijian Zha.
